# A Non-targeted Proteomics Newborn Screening Platform for Inborn Errors of Immunity

**DOI:** 10.1007/s10875-024-01821-7

**Published:** 2024-10-25

**Authors:** Hirofumi Shibata, Daisuke Nakajima, Ryo Konno, Atsushi Hijikata, Motoko Higashiguchi, Hiroshi Nihira, Saeko Shimodera, Takayuki Miyamoto, Masahiko Nishitani-Isa, Eitaro Hiejima, Kazushi Izawa, Junko Takita, Toshio Heike, Ken Okamura, Hidenori Ohnishi, Masataka Ishimura, Satoshi Okada, Motoi Yamashita, Tomohiro Morio, Hirokazu Kanegane, Kohsuke Imai, Yasuko Nakamura, Shigeaki Nonoyama, Toru Uchiyama, Masafumi Onodera, Ryuta Nishikomori, Osamu Ohara, Yusuke Kawashima, Takahiro Yasumi

**Affiliations:** 1 Present Address: Department of Pediatrics, Kyoto University Graduate School of Medicine, 54 Shogoin-Kawahara-Cho, Sakyo-Ku, Kyoto, 606-8507 Japan; 2 Present Address: Department of Applied Genomics, Kazusa DNA Research Institute, 2-5-23 Kazusa-Kamatari, Kisarazu, 292-0818 Japan; 3 School of Life Sciences, Tokyo University of Pharmacy and Life Sciences, Tokyo, Japan; 4 Department of Dermatology, Faculty of Medicine, Yamagata University, Yamagata, Japan; 5 Department of Pediatrics, Gifu University Graduate School of Medicine, Gifu, Japan; 6 Department of Pediatrics, Graduate School of Medical Sciences, Kyushu University, Fukuoka, Japan; 7 Department of Pediatrics, Hiroshima University Graduate School of Biomedical and Health Sciences, Hiroshima, Japan; 8 Department of Pediatrics and Developmental Biology, Graduate School of Medical and Dental Sciences, Tokyo Medical and Dental University (TMDU), Tokyo, Japan; 9 Department of Child Health and Development, Graduate School of Medical and Dental Sciences, Tokyo Medical and Dental University (TMDU), Tokyo, Japan; 10 Department of Pediatrics, National Defense Medical College, Tokorozawa, Japan; 11 Department of Human Genetics, National Center for Child Health and Development, Tokyo, Japan; 12 Gene & Cell Therapy Promotion Center, National Center for Child Health and Development, Tokyo, Japan; 13 Department of Pediatrics and Child Health, Kurume University School of Medicine, Kurume, Japan; 14 Japan Environment and Children’s Study Kyoto Regional Center, Kyoto University Graduate School of Medicine, Kyoto, Japan

**Keywords:** Newborn screening, Dried blood spot, Non-targeted proteomics

## Abstract

**Purpose:**

Newborn screening using dried blood spot (DBS) samples for the targeted measurement of metabolites and nucleic acids has made a substantial contribution to public healthcare by facilitating the detection of neonates with genetic disorders. Here, we investigated the applicability of non-targeted quantitative proteomics analysis to newborn screening for inborn errors of immunity (IEIs).

**Methods:**

DBS samples from 40 healthy newborns and eight healthy adults were subjected to non-targeted proteomics analysis using liquid chromatography-mass spectrometry after removal of the hydrophilic fraction. Subsequently, DBS samples from 43 IEI patients were analyzed to determine whether patients can be identified by reduced expression of disease-associated proteins.

**Results:**

DBS protein profiling allowed monitoring of levels of proteins encoded by 2912 genes, including 1110 listed in the Online Mendelian Inheritance in Man database, in healthy newborn samples, and was useful in identifying patients with IEIs by detecting reduced levels of disease causative proteins and their interacting proteins, as well as cell-phenotypical alterations.

**Conclusion:**

Our results indicate that non-targeted quantitative protein profiling of DBS samples can be used to identify patients with IEIs and develop a novel newborn screening platform for genetic disorders.

**Supplementary Information:**

The online version contains supplementary material available at 10.1007/s10875-024-01821-7.

## Introduction

Dried blood spot (DBS) samples, comprising spotted blood dried in filter paper, are widely used for pre-symptomatic disease screening because of their simplicity in terms of sampling, shipping, and storage. Since Dr. Guthrie introduced the first screening test for phenylketonuria [1], newborn screening (NBS) programs using DBS samples have made considerable contributions to public healthcare. NBS can save neonates with serious inborn disorders from developing devastating symptoms, by introducing appropriate therapeutic intervention before disease onset, which can improve their long-term prognosis. Notably, the emergence of tandem mass spectrometry has enabled use of a single platform to simultaneously screen for a wide range of inborn errors of metabolism (IEMs), playing a critical role in the spread of NBS [2] and serving as a successful example of the power of non-targeted measurement platforms for pre-symptomatic screening for inherited diseases, followed by confirmatory diagnosis by genetic analyses.

Although NBS for IEMs can be achieved by assessing metabolic function through monitoring activity of metabolites or metabolic enzymes, direct measurement of molecular function is frequently too complex and infeasible in the context of NBS for other inherited conditions. As recent advances in DNA sequencing technology have dramatically improved cost-performance and throughput, the use of genome-wide sequencing has been proposed as a new alternative modality for NBS [3]; however, there remain many technical, clinical, ethical, and social issues to be addressed before implementation of genome sequencing for NBS [4, 5]. In addition, concerns regarding the use of genome sequencing persist, due to an inability to predict gene product functionality from structural genetic information with high accuracy; in the absence of accumulated in-depth genotype–phenotype data, gene structure information cannot be linked with clinical phenotypes. Indeed, Adhikari et al. reported that exome sequencing was not sufficiently sensitive or specific as a primary screen for use in NBS for IEM, while it is useful as a second-tier test for some infants who test positive in first-tier screening, assisted by tandem mass spectrometry [6].

As the majority of inherited diseases occur due to loss or gain of protein function, we hypothesized that measurement of the quality and/or quantity of proteins encoded by causative genes could represent a new modality for NBS with advantages over structural gene analyses. We recently reported that proteome analysis of peripheral blood mononuclear cells can complement genetic analysis in identifying the causes of inborn errors of immunity (IEIs) in undiagnosed patients [7]. Here, we explore whether proteome analysis of DBS samples can contribute to the diagnosis/screening of IEIs and other genetic disorders. Targeted quantitative measurement of proteins of interest in DBS have been reported as test trials [8, 9]; however, until recently, cost and throughput issues have not been surmounted. Recent advances in proteome technology have allowed us to address these issues; for example, advanced liquid chromatography-assisted mass spectrometry (LC–MS/MS) in data-independent acquisition (DIA) mode facilitates highly sensitive quantification of protein levels [10, 11]. Furthermore, a non-targeted method for in-depth DBS protein profiling with high reproducibility has also been developed [12]. These technical advances have allowed us to overcome the obstacles impeding progress in proteome analysis-based NBS.

In this context, the aims of the present study were to evaluate whether non-targeted proteomics analysis of newborn DBS by LC–MS/MS could be used to identify and quantify disease-causing or -related proteins, thereby clarifying the molecular phenotypes underlying genetic disorders at a pre-symptomatic stage. For this purpose, DBS samples from 40 healthy newborns were first subjected to non-targeted proteomics analysis, to assess their consistency and reliability for use in protein profiling by comparison with similar data generated from healthy adult samples. Subsequently, DBS samples from patients with IEIs, as a representative disease spectrum primarily caused by defects in genes expressed in blood, were analyzed to determine whether patients with these conditions can be identified by quantitative protein profiling. Our results indicate that non-targeted DBS proteomics can detect changes in molecular and cellular phenotypes underlying many IEIs, indicating that this approach may be useful for newborn diagnosis and/or screening for genetic diseases.

## Methods

### Patients and Samples

DBS samples on Guthrie cards from 40 healthy newborns used to screen for metabolic disorders were collected, as were samples from 8 healthy adults, and 45 samples from 43 patients with 12 IEI diseases. Of the IEI samples, 38 were obtained after disease onset and stored at their respective institutions for research purposes, and the remaining FHL type 3 (n = 5) and CHS (n = 1) samples were obtained pre-symptomatically for use in public health screening for metabolic diseases. Detailed sample information is presented in Table 1.

**Table 1:** Details of 45 dried blood spot samples from 43 patients with inborn errors of immunity analyzed in the current study

Patient	Sample	Age at sampling (months)	Disease	Causative Gene	Causative Protein	Mutation		Protein levels encoded by the mutated gene (relative to ACTB)		
						Allele 1	Allele 2	Patient	Healthy newborns	Healthy adults
1	1	3	FHL type 3	*UNC13D*	Munc13-4	c.1596 + 1G > C	c.1255delC, p.Leu419Serfs*23	0.143	Mean, 10.9; SD, 2.6; Min, 5.9	Mean. 8.2; SD, 3.2; Min, 3.3
2	2	266				c.1596 + 1G > C	c.1240C > T, p.Arg414Cys	0.192		
3	3^b^	0				c.1596 + 1G > C	c.118-308C > T	0.357		
4	4^b^	0				c.1596 + 1G > C	c.1545-2A > G	0.182		
5	5	1				c.754-1G > C	c.1596 + 1G > C	0.132		
	6^b^	0						0.120		
6	7	4				c.1849-1G > C	c.1849-1G > C	0.270		
	8^b^	0						0.360		
7^a^	9	0				c.1596 + 1G > C	c.1596 + 1G > C	0.280		
8	10^b^	0				c.754-1G > C	c.1596 + 1G > C	0.190		
9	11	3	FHL type 5	*STXBP2*	Munc18-2	c.1197delC, p.Ala400Profs*18	c.1430C > T, p.Pro477Leu	1.120	Mean, 10.9; SD, 2.1; Min, 4.0	Mean, 7.8; SD, 2.1; Min, 4.8
10	12	1	FHL type 2	*PRF1*	perforin	c.658G > A, p.Gly220Ser	c.1090_1091delCT, p.Leu364Glufs*93	0.290	Mean, 0.33; SD, 0.28; Min, 0.28	Mean, 0.30; SD, 0.35; Min, 0.044
11	13	2				c.853_855delAAG, p.Lys285del	c.1090_1091delCT, p.Leu364Glufs*93	0.330		
12	14	9	HPS type 2	*AP3B1*	AP3B1	c.2546T > A, p.Leu849X	c.1122_1123insAG, p.Phe375Serfs*11	0.183	Mean, 2.93; SD, 0.88; Min, 1.08	Mean, 0.91; SD, 0.25; Min, 0.58
13	15	102				c.364C > T, p.Arg122X	c.2810-1G > T	1.080		
14	16	38				c.2546T > A, p.Leu849X	c.188T > A, p.Met63Lys	0.439		
15	17	19	CGD	*CYBB*	p91-phox	c.1528_1529delTT, p.Leu510Valfs*8	NA	0.010	Mean, 2.63; SD, 1.36; Min, 0.72	Mean, 0.78; SD, 0.51; Min, 0.30
16	18	318				c.121dupT, p.Tyr41Leufs*62	NA	0.020		
17	19	87				c.1031C > T, p.Ser344Phe	NA	0.010		
18	20	62				c.252G > A, p.Ala84Ala	NA	0.024		
19	21	48				c.810G > A, p.Trp270X	NA	0.015		
20	22	105				c.271C > T, p.Arg91X	NA	0.058		
21	23	213	CGD	*CYBA*	p22-phox	c.70G > A, p.Gly24Arg	c.7C > T, p.Gln3X	Below detection limit	Mean, 7.1; SD, 4.1; Min, 1.9	Mean, 3.5; SD, 1.4; Min, 1.7
22	24	132	CGD	*NCF1*	p47-phox	c.75_76delGT, p.Tyr26Hisfs*26	c.75_76delGT, p.Tyr26Hisfs*26	0.010	Mean, 0.18; SD, 0.099; Min, 0.025	Mean, 0.10; SD, 0.062; Min, 0.057
23	25	12	WAS	*WAS*	WASP	c.777 + 3_777 + 6delGAGT	NA	Below detection limit	Mean, 0.098; SD, 0.039; Min, 0.022	Mean, 0.056; SD, 0.025; Min, 0.024
24	26	4				c.132 + 1G > T	NA	Below detection limit		
25	27	70				c.982delC, p.Arg328Glyfs*117	NA	0.011		
26	28	226				c.961C > T, p.Arg321X	NA	Below detection limit		
27	29	6				c.1075C > A, p.Pro359Thr	NA	Below detection limit		
28	30	26	XLA	*BTK*	BTK	c.1574G > A, p.Arg525Gln	NA	0.005	Mean, 0.60; SD, 0.16; Min, 0.25	Mean, 0.39; SD, 0.17; Min, 0.26
29	31	36				c.1856C > T, p.Pro619Leu	NA	0.004		
30	32	20				c.862C > T, p.Arg288Trp	NA	0.017		
31	33	386				c.1921C > T, p.Arg641Cys	NA	0.029		
32	34	113				c.902-904delAAG, p.Glu301del	NA	0.055		
33	35	456				c.95T > C, p.Leu32Ser	NA	0.002		
34	36	255				c.1856C > T, p.Pro619Leu	NA	0.035		
35	37	0	SCID	*ADA*	ADA1	c.218 + 2T > G	c.218 + 2 T > G	0.040	Mean, 0.075; SD, 0.054; Min, 0.014	Mean, 0.18; SD, 0.096; Min, 0.047
36	38	3				c.218 + 2T > G	c.632G > A, p.Arg211His	0.108		
37	39	0	X-SCID	*IL2RG*	IL2Rγ	c.865C > T, p.Arg289X	NA	Below detection limit	NA	NA
38	40	2				c.374A > G, p.Tyr125Cys	NA	Below detection limit		
39	41^b^	0	CHS	*LYST*	LYST	c.1673dupT, p.Leu558Phefs*22	c.5506C > T, p.Arg1836X	0.003	Mean, 0.056; SD, 0.035; Min, 0.01	Mean, 0.025; SD, 0.018; Min, 0.075
40	42	213				c.3393 + 1G > T	c.3393 + 1G > T	0.016		
41	43	373				c.5541_5542delAA, p.Arg1848Serfs*3	unknown	0.012		
42	44	346				c.5541_5542delAA, p.Arg1848Serfs*3	unknown	0.009		
43	45	346				c.5541_5542delAA, p.Arg1848Serfs*3	unknown	0.026		

*FHL* familial hemophagocytic lymphohistiocytosis, *HPS* Hermansky-Pudlak syndrome, *CGD* chronic granulomatous disease, *WAS* Wiskott-Aldrich syndrome, *XLA* X-linked agammaglobulinemia, *X-SCID* X-linked SCID, *CHS* Chédiak-Higashi syndrome, *NA* not applicable

^a^Fetal-onset case

^b^Samples were obtained from pateints before symptoms appeared and have been used for public screening of metabolic diseases

### Preparation and Digestion of the Insoluble Protein Fraction

A disc (3.2 mm diameter) punched from a DBS was suspended in 1 mL of Na_2_CO_3_ solution and vigorously shaken in the presence of a 5 mm zirconia bead (Tomy Seiko, Tokyo, Japan) using a Tissue Lyser (Qiagen, Hilden, Germany) oscillating at 25 Hz for 5 min at room temperature. The remaining disc was removed by centrifugation (3,000 × g, 3 min, 4 °C) and the supernatant (approximately 850 μL) was centrifuged (17,400 g, 15 min, 4 °C). The precipitate was resuspended in 200 μL 100 mM sodium carbonate aqueous solution by vigorous vortexing at room temperature. After centrifugation (17,400 g, 5 min, 4 °C), the supernatant was completely removed and the precipitate dissolved in 100 μL 12 mM sodium deoxycholate, 12 mM N-lauroylsarcosine, and 100 mM Tris–Cl pH 8.0 by vigorous vortexing for 1 min, followed by sonication using Bioruptor II (CosmoBio, Tokyo, Japan) on high power mode for 10 min in 30 s on/30 s off cycles. A 500 ng aliquot of trypsin/lys-C mix (Promega, Madison, WI) was added to each protein extract and samples were digested overnight at 37 °C. After precipitation, detergent was removed by acidification with 30 μL 5% trifluoroacetic acid, followed by sonication with a Bioruptor II in high power mode for 5 min in 30 s on/30 s off cycles. The mixture was shaken for 5 min and centrifuged (15,000 g, 5 min). The supernatant was desalted using GL-Tip SDB (GL Sciences Inc., Tokyo, Japan), according to the manufacturer’s instructions, and dried using a centrifugal evaporator (miVac Duo concentrator, Genevac, Ipswich, UK). Dried peptides were redissolved in 3% ACN, 0.1% formic acid.

### LC–MS/MS

Digested peptides were directly injected onto a 75 μm × 20 cm PicoFrit emitter (New Objective, Woburn, MA) packed in-house with C18 core–shell particles (CAPCELL CORE MP 2.7 μm, 160 Å material; Osaka Soda Co., Ltd., Osaka, Japan) at 50 °C and peptides separated using an UltiMate 3000 RSLCnano LC system (Thermo Fisher Scientific, Waltham, MA), with an 80 min gradient at 100 nL/min. Peptides eluted from the column were analyzed on an Orbitrap Exploris 480 Mass Spectrometer (Thermo Fisher Scientific) for overlapping window DIA-MS [11, 13]. MS1 spectra were collected in the range, 495–785 m/z, at 30,000 resolution, to set an automatic gain control target of 3 × 10^6^ and maximum injection time of “auto”. MS2 spectra were collected in the range, 200–1800 m/z, at 30,000 resolution to set an automatic gain control target of 3 × 10^6^, maximum injection time of “auto”, and stepped normalized collision energies of 22%, 26%, and 30%. The MS2 isolation width was set to 4 m/z, and overlapping window patterns of 500–780 m/z used for window placements optimized by Skyline (20.2.0.34, University of Washington, Seattle, WA) [14].

### MS Data Analysis

MS files were compared with human spectral libraries using Scaffold DIA (2.2.1, Proteome Software, Inc., Portland, OR). Human spectral libraries were generated from the human protein sequence database (UniProt id UP000005640, reviewed, canonical) using Prosit (https://www.proteomicsdb.org/prosit/) [15, 16]. Scaffold DIA search parameters were as follows: experimental data search enzyme, trypsin; maximum missed cleavage sites, 1; precursor mass tolerance, 9 ppm; and fragment mass tolerance, 9 ppm. The protein identification threshold was set at < 1% for both peptide and protein false discovery rates. Proteins and peptides were quantified using the EncyclopeDIA algorithm in Scaffold DIA [17].

### Prediction of Effects of Missense Mutations on Protein Stability

Crystal structures of the proteins encoded by the disease-causing genes ADA, CYBA, CYBB, IL2RG, and STXBP2 were downloaded from Protein Data Bank (https://pdbj.org/) (PDB codes: 3iar for ADA; 8gz3 for CYBA and CYBB; 5m5e for IL2RG; 4cca for STXBP2). Where there were no experimentally determined structures for proteins with mutated residues (namely, AP3B1, PRF1, UNC13D, and WAS), protein model structures were obtained from the AlphaFold Protein Structure Database (https://alphafold.ebi.ac.uk/). Computational predictions of the impacts of missense mutations on protein stability were evaluated using FoldX, which is a state-of-the art method for stability estimation based on the three-dimensional protein structures [18]. If the total energy stability change value of the mutant protein was > 1.58 kcal/mol, the missense mutation was predicted to affect protein stability, as described in a previous report [19].

### Statistical Analysis

Protein expression levels were standardized using those of β-actin (%β-actin). Exploratory analysis, including log_2_ transformation, multiple scatter plotting, calculation of Pearson correlation coefficient values between two sets of data, data imputation (width = 0.3, Down shift = 2.4), Z-score normalization, and hierarchical comparison were performed using the Perseus software platform (https://maxquant.net/perseus/). Volcano plots were created by multiple t test using the two-stage step up method of Benjamini, Krieger, and Yekutieli in GraphPad Prism (version 9.0.2) software (GraphPad Software, Inc., La Jolla, CA). DEPs between two groups were selected for further analysis using a cutoff of adjusted P value (q value) < 0.01 and fold-change ≥ 2. Statistical differences among groups were analyzed using one-way ANOVA on ranks (Kruskal–Wallis test), followed by Dunn’s multiple comparisons test using GraphPad Prism. A P value < 0.05 was considered significant.

### Study Approval

The study protocol, which conformed to the principles of the Declaration of Helsinki, was approved by the ethics committee of Kyoto University Hospital (G1118, G1278, and R2073). Written informed consent was obtained from all participants or their legal guardians.

### Data Availability

Table S1 and S2 are also available at 10.5281/zenodo.13890745. All figures were generated using the data in Table S1.

## Results

### Comparison of Protein Profiles from Healthy Newborn and Healthy Adult Samples

**Fig. 1:**
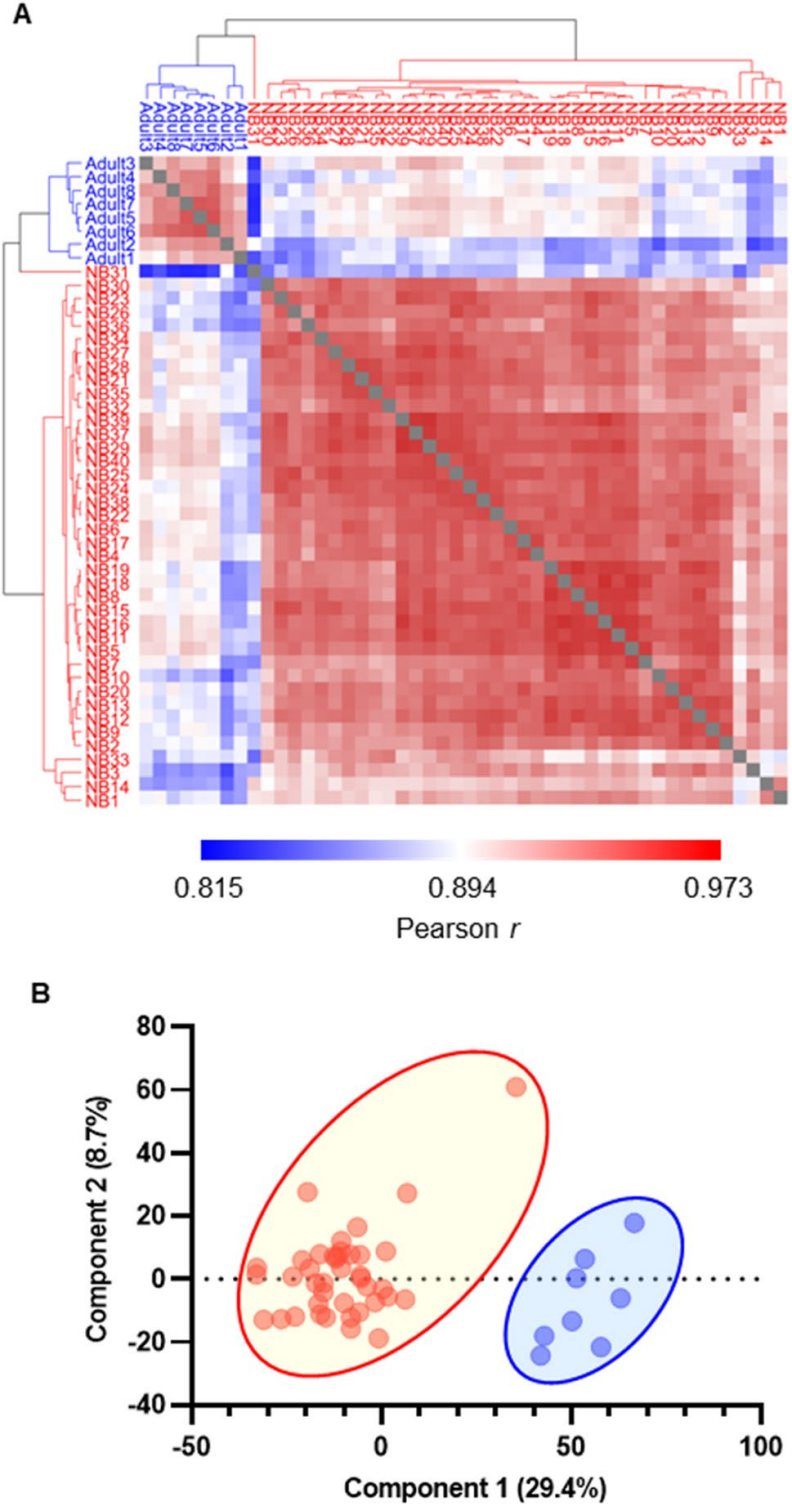
Proteomics analysis distinguishes healthy newborn dried blood spot (DBS) samples from those of adults. **A** Clustered heatmap of Pearson correlation coefficient values between all healthy newborn and adult DBS sample pairs. Dark red denotes higher correlation and dark blue denotes lower correlation (Pearson r = 0.82–0.96). **B** Principal component analysis of healthy newborn and adult DBS sample pairs. Red denotes newborn (NB) and blue denotes adult DBS samples

**Fig. 2:**
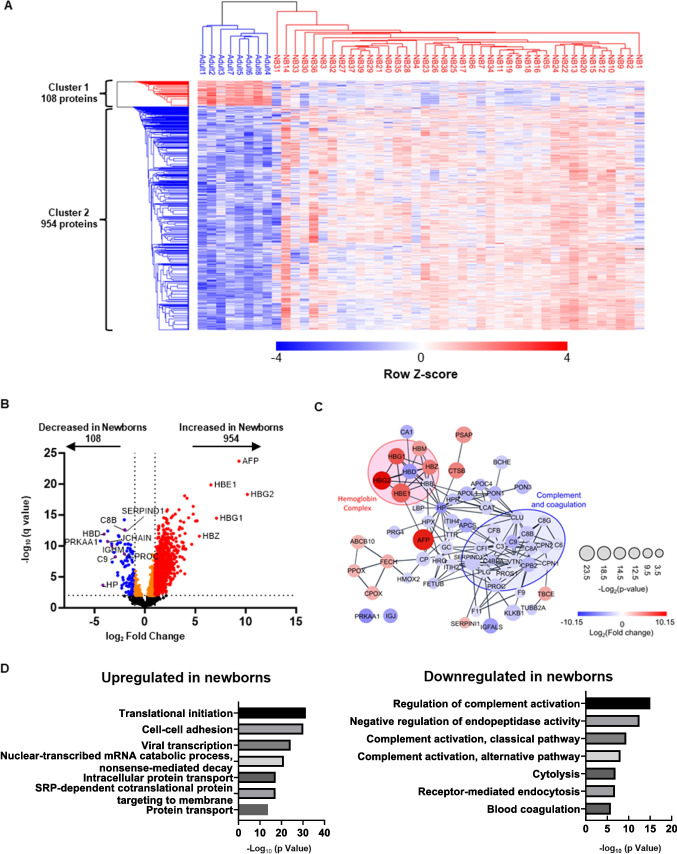
Proteins differentially expressed between healthy newborn and adult samples. **A** Heatmap of hierarchical clustering of all differentially expressed proteins (DEPs) detected by comparison between healthy newborn and adult samples. Red, upregulated DEPs; blue, downregulated DEPs. **B** Volcano plot showing DEPs in dried blood spot samples from healthy newborns compared with those from healthy adults. **C** Protein–protein interaction network analysis illustrating the top 100 upregulated and downregulated proteins (q-value < 0.05), contrasting healthy newborns and adults; network generated in Cytoscape using the STRING database. Node size corresponds to P value, while colors (red and blue) signify DEPs; red and blue circles represent the 'Hemoglobin complex' and 'Complement and coagulation' Gene Ontology (GO) terms, respectively. **D** GO enrichment analysis of DEPs. Bar charts showing the top 10 GO terms for biological processes upregulated (left) and downregulated (right) in newborn samples relative to adult samples

LC–MS/MS and non-targeted deep proteome analyses of DBS samples from 40 healthy newborns enabled the identification and quantification of 3587 proteins within the identification threshold (Table S1). These data were filtered to include only proteins with ≥ 2 identified peptides, resulting in a total of 2912 proteins that could be quantified within measurable ranges in all newborn samples. Of these, 1110 proteins encoded by genes listed in the Online Mendelian Inheritance in Man (OMIM) database were subjected to analysis after standardization based on β-actin levels in the same sample (Table S2). We also analyzed samples from eight healthy adults. Correlation analysis indicated that samples in the newborn and adult DBS groups showed high within-group coefficient values (Pearson r = 0.849–0.973 and 0.898–0.962, respectively). Next, we conducted multiple evaluation of Pearson’s correlation coefficient values between the two sample types, along with principal component analysis, and the results showed that newborn samples clustered separately from adult samples (Fig. 1A and B). Detailed analysis of protein levels to search for differentially expressed proteins (DEPs) identified 954 upregulated and 108 downregulated proteins in newborn, relative to adult, samples (Fig. 2A). Upregulated proteins included components of fetal and embryonic hemoglobin (*HBG1/HBG2* and *HBE1/HBZ*, respectively) and alfa fetoprotein (*AFP*). Downregulated proteins included antithrombin III (*SERPINC1*), heparin cofactor 2 (*SERPIND1*), protein C (*PROC*), haptoglobin (*HP*), Hb subunit delta (*HBD*), immunoglobulin heavy constant mu (*IGHM*), and joining chain of multimeric IgA and IgM (*JCHAIN*) (Fig. 2B, C and Fig. S1). In Gene Ontology (GO) analysis of DEPs, translational initiation and regulation of complement activation pathways were ranked as highly enriched for proteins upregulated and downregulated in newborn relative to adult samples, respectively (Fig. 2D and E). These results are generally consistent with previous reports [20, 21] and collectively indicate the reliability of the current method for non-targeted quantitative evaluation of proteins in DBS samples.

### Proteins Causative of Various Genetic Disorders can be Quantified in Neonatal DBS Samples

**Fig. 3:**
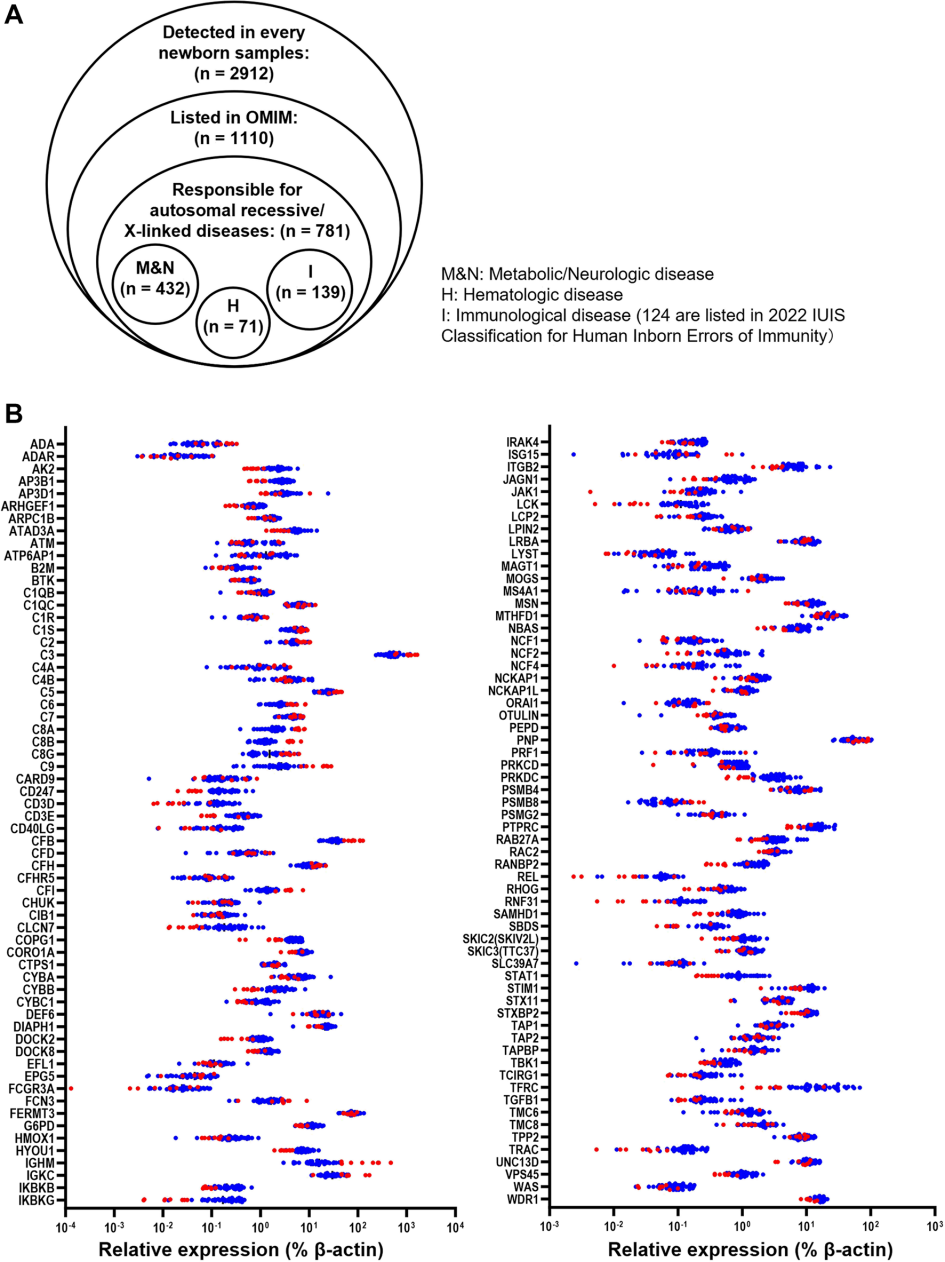
Quantification of proteins responsible for various genetic disorders in neonatal dried blood spot samples. **A** Venn diagram of proteins identified in DBS samples. OMIM, Online Mendelian Inheritance in Man. **B** Levels of 124 proteins listed in the 2022 International Union of Immunological Societies classifications of inborn errors of immunity in DBS samples from healthy newborns (blue) and healthy adults (red)

Next, we considered which diseases are feasible potential targets for diagnosis/screening by DIA-LC–MS/MS using DBS samples. Diseases with autosomal recessive or X-linked inheritance were identified as primary targets; as diseases with autosomal dominant inheritance are caused by anomaly of a single allele, while the other allele remains intact, which would be expected to minimally affect protein levels, they were not considered ideal for screening using this approach. Of the 1110 identified proteins listed in OMIM, 781 were related to diseases with autosomal recessive or X-linked inheritance, of which, 432, 71, and 139 proteins were categorized as metabolic/neurological, hematological, and immunological diseases, respectively (Fig. 3A). Of the 139 proteins related to immunological diseases, 124 were included as causative molecules in the latest list of IEIs (Table S2) and most of these were distributed similarly in healthy newborn and adult samples (Fig. 3B). To explore the possibility of direct detection of changes in protein levels caused by genetic mutations and their use for diagnosis/screening of genetic disorders, we next focused our efforts on characterization of DBS protein levels in samples from patients with IEIs (Table 1), as most IEI causative proteins are abundantly expressed in peripheral blood.

### Primary Effect of IEI Gene Mutations on Levels of their Encoded Proteins

**Fig. 4:**
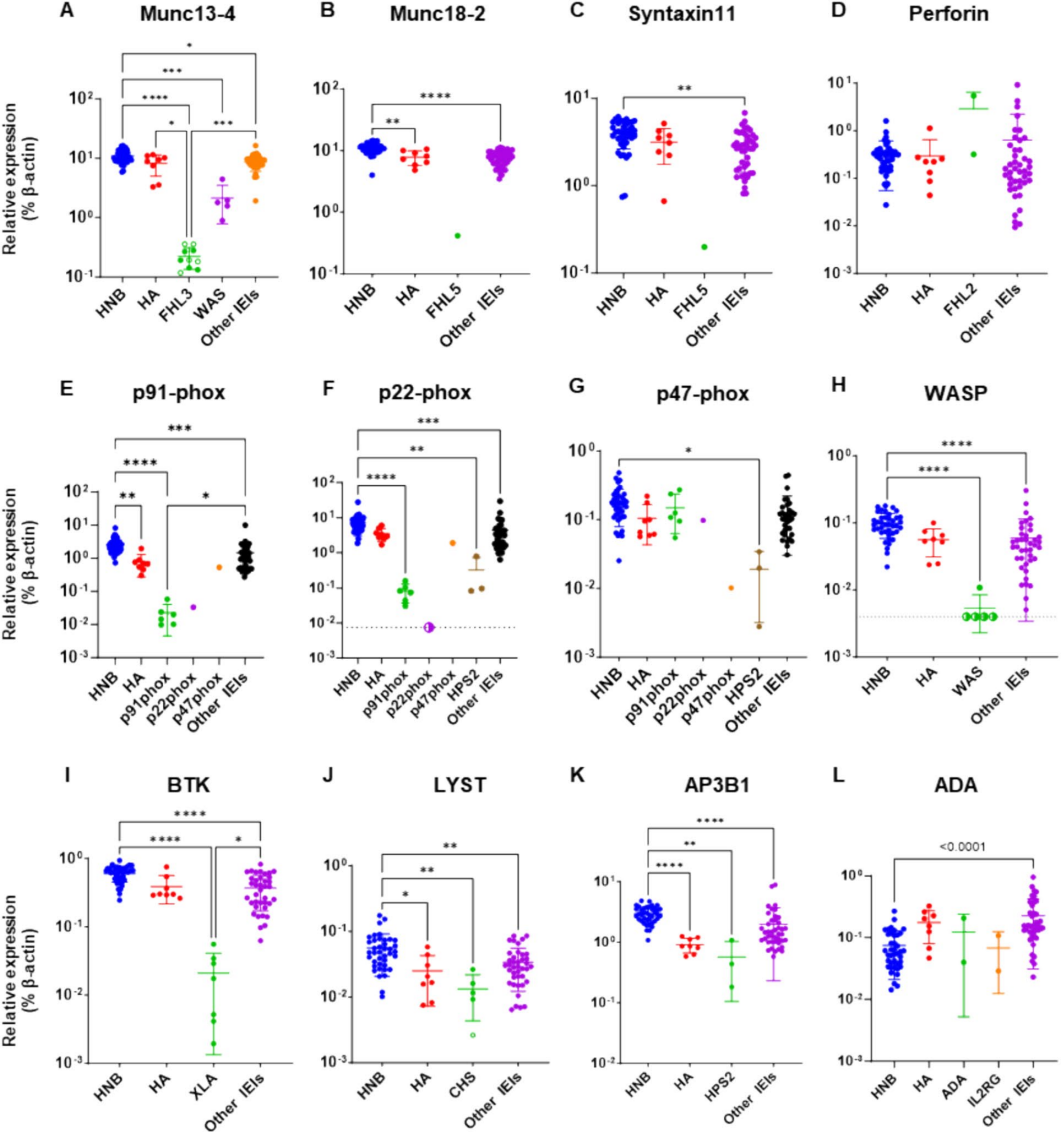
Expression of disease-responsible proteins in samples from patients with inborn errors of immunity. Proteomics evaluation of disease-responsible proteins in samples from healthy newborns (HNB), healthy adults (HA), and patients with IEIs, including: familial hemophagocytic lymphohistiocytosis (FHL) type 2, 3, and 5 (FHL2: *n* = 2, FHL3: *n* = 8, and FLH5: *n* = 1, respectively); p91-phox, p22-phox, and p47-phox deficiencies (*n* = 6, 1, and 1, respectively); Hermansky-Pudlak syndrome type 2 (HPS2: *n* = 3); Wiskott-Aldrich syndrome (WAS: *n* = 5); X-linked agammaglobulinemia (XLA: *n* = 7); Chédiak-Higashi syndrome (CHS: *n* = 5); adenosine deaminase deficiency (ADA: *n* = 2); common γ chain deficiency (IL2RG: *n* = 2). All data were standardized relative to the expression of β-actin in the same sample. Levels of **(A)** Munc13-4, **(B)** Munc18-2, **(C)** Syntaxin-11, **(D)** perforin, **(E)** p91-phox, **(F)** p22-phox, **(G)** p47-phox, **(H)** WASP, **(I)** BTK, **(J)** LYST, **(K)** AP3B1, and **(L)** ADA in samples from HNB, HA, and patients with IEIs. Open symbols, samples obtained from pre-symptomatic newborn patients with IEIs; half-closed symbols, results under the limit of detection plotted at approximate lower detection limits. Statistical differences among groups were analyzed using one-way ANOVA on ranks (the Kruskal–Wallis test), followed by Dunn’s multiple comparisons. Data represent the mean ± SD. *P < 0.05, **P < 0.01, ***P < 0.001, ****P < 0.0001

First, we evaluated whether levels of IEI disease-causing proteins were exclusively reduced in patient samples and suitable for use in disease diagnosis. All 10 samples from patients with familial hemophagocytic lymphohistiocytosis (FHL) type 3, including five obtained before and five obtained after the onset of disease, showed significant reductions in Munc13-4 levels compared to control samples (Fig. 4A and Fig. S2). This finding is consistent with reports showing that FHL type 3 can be reliably diagnosed by assessing intra-platelet Munc13-4 levels [22, 23]. A similar result was observed in a patient with FHL type 5, in that Munc18-2 levels were markedly lower in the patient than in control samples (Fig. 4B). By contrast, perforin levels did not differ significantly between patients with FHL type 2 and controls (Fig. 4D), although flow cytometry showed that perforin levels were reduced in natural killer cells from patients (Fig. S3). Analysis of protein profiles in patients with other IEIs showed that levels of p91-phox, Wiskott-Aldrich syndrome protein (WASP), and Bruton’s tyrosine kinase (BTK) in samples from patients with p91-phox deficiency, Wiskott-Aldrich syndrome (WAS), and X-linked agammaglobulinemia (XLA), respectively, were markedly lower than those in control samples (Fig. 4E, H, and I). A sample from a patient with p22-phox deficiency also showed markedly reduced levels of its causative protein (Fig. 4F). Interestingly, p22-phox levels paralleled those of p91-phox in this patient (Fig. 4E and F), likely reflecting the tight interaction between these molecules [24, 25]. A similar effect was observed in an FHL type 5 sample, in that Syntaxin-11 levels were reduced in parallel with those of Munc18-2 (Fig. 4B and C). Levels of the causative protein were also lower in sample from a patient with p47-phox deficiency than those in healthy controls; however, they were only slightly below the normal range (Fig. 4G). Samples from patients with Hermansky-Pudlak syndrome (HPS) type 2 also had reduced p47-phox protein levels (Fig. 4G). By contrast, levels of lysosomal trafficking regulator (LYST), adaptor related protein complex 3 subunit beta 1 (AP3B1), and adenosine deaminase (ADA) in patients with Chédiak-Higashi syndrome (CHS), HPS type 2, and ADA deficiency, respectively, did not differ significantly from those in control samples (Fig. 4J–L). Levels of common γ chain (IL2RG) were below the limit of detection in every sample. Absolute expression levels of the disease-causing protein in every patient, together with those in healthy controls, are summarized in Table 1.

We next determined whether the effect of a gene mutation on its protein level can be reliably predicted by in silico analysis. Table 2 shows the results of mutation effect predictions, together with the observed protein levels. As missense mutations often affect protein abundance by destabilizing protein structures [26], we performed computational prediction of the impact of missense mutations on protein stability using FoldX, a state-of-the art method for stability estimation based on the three-dimensional protein structures [18]. Of the 14 missense mutations identified in patients, 10 led to reduced protein abundance. Of those mutations leading to protein loss, 8 (80%) were successfully predicted to be destabilized, based on a total energy change threshold of > 1.58 kcal/mol for affecting protein stability upon a missense mutation [19]; however, two mutations, namely p.Pro359Thr in WASP and p.Arg525Gln in BTK, were falsely predicted to result in protein loss. Further, three mutations, namely p.Gly220Ser in perforin, p.Met63Lys in AP3B1, and p.Arg211His in ADA, were predicted to destabilize the proteins, while proteins harboring the mutations showed no loss of abundance in our experiments. These results indicate that use of an in silico approach is not sufficiently reliable, in terms of sensitivity and specificity, to evaluate the impact of missense mutations on protein abundance.

**Table 2:** Predicted and observed levels of mutated proteins

Disease	Causative gene	Mutated allele	Mutation type	Pathogenicity determined by Mutation effect predictor^a^	NMD probability^b^	Predicted structural stability^c^	Observed protein loss
FHL type 3	*UNC13D*	c.1240C > T, p.Arg414Cys	Missense mutation	Deleterious (aggregated score, 0.856)		3.28	Yes
		c.1255delC, p.Leu419Serfs*23	Frameshift		NMD		Yes
		c.1596 + 1G > C	Splicing anomaly				Yes
		c.118-308C > T	Transcriptional dysregulation		Predicted benign		Yes
		c.754-1G > C	Splicing anomaly		Exon skip 105 bp		Yes
		c.1849-1G > C	Splicing anomaly		Exon skip 144 bp		Yes
		c.1545-2A > G	Splicing anomaly		Exon skip 54 bp		Yes
FHL type 5	*STXBP2*	c.1430C > T, p.Pro477Leu	Missense mutation	Deleterious (aggregated score, 0.857)		4.29	Yes
		c.1197delC, p.Ala400Profs*18	Frameshift		NMD		Yes
FHL type 2	*PRF1*	c.658G > A, p.Gly220Ser	Missense mutation	Deleterious (aggregated score, 0.98)		14.1	No
		c.853_855delAAG, p.Lys285del	deletion	benign			No
		c.1090_1091delCT, p.Leu364Glufs*93	Frameshift		NMD escaping		No
HPS type 2	*AP3B1*	c.188T > A, p.Met63Lys	Missense mutation	Uncertain (aggregated score, 0.67)		5.13	No
		c.1122_1123insAG, p.Phe375Ser*11	Frameshift		NMD		Yes
		c.2546T > A, p.Leu849X	Nonsense mutation		NMD		Yes
		c.364C > T, p.Arg122X	Nonsense mutation		NMD		Reduced but detectable
		c.2810-1G > T	Splicing anomaly		Exon skip 85 bp		Reduced but detectable
CGD	*CYBB*	c.1031C > T, p.Ser344Phe	Missense mutation	Deleterious (aggregated score, 0.99)		12.5	Yes
		c.1528_1529delTT, p.Leu510Valfs*8	Frameshift		NMD		Yes
		c.121dupT, p.Tyr41Leufs*62	Frameshift		NMD		Yes
		c.810G > A, p.Trp270X	Nonsense mutation		NMD		Yes
		c.271C > T, p.Arg91X	Nonsense mutation		NMD		Yes
		c.252G > A, p.Ala84Ala	Splicing anomaly	Deleterious (aggregated score, 0.80)	splicing anomaly		Yes
CGD	*CYBA*	c.70G > A, p.Gly24Arg	Missense mutation	Deleterious (aggregated score, 0.87)		6.54	Yes
		c.7C > T, p.Gln3X	Nonsense mutation		NMD escaping		Yes
CGD	*NCF1*	c.75_76delGT, p.Tyr26Hisfs*26	Frameshift		NMD escaping		Reduced but detectable
WAS	*WAS*	c.1075C > A, p.Pro359Thr	Missense mutation	Uncertain (aggregated score, 0.54)		0.37	Yes
		c.982delC, p.Arg328Glyfs*117	Frameshift		NMD		Reduced but detectable
		c.961C > T, p.Arg321X	Nonsense mutation		NMD		Yes
		c.777 + 3_777 + 6delGAGT	Splicing anomaly				Yes
		c.132 + 1G > T	Splicing anomaly				Yes
XLA	*BTK*	c.95T > C, p.Leu32Ser	Missense mutation	Deleterious (aggregated score, 0.99)		5.26	Yes
		c.862C > T, p.Arg288Trp	Missense mutation	Deleterious (aggregated score, 0.88)		1.81	Yes
		c.1574G > A, p.Arg525Gln	Missense mutation	Deleterious (aggregated score, 0.88)		0.02	Yes
		c.1856C > T, p.Pro619Leu	Missense mutation	Deleterious (aggregated score, 0.87)		6.19	Yes
		c.1921C > T, p.Arg641Cys	Missense mutation	Deleterious (aggregated score, 0.88)		2.95	Yes
		c.902-904delAAG, p.Glu301del	Deletion				Yes
SCID	*ADA*	c.632G > A, p.Arg211His	Missense mutation	Deleterious (aggregated score, 0.99)		9.20	No
		c.218 + 2T > G	Splicing anomaly				No
X-SCID	*IL2RG*	c.374A > G, p.Tyr125Cys	Missense mutation	Deleterious (aggregated score, 0.86)		3.36	ND
		c.865C > T, p.Arg289X	Nonsense mutation		NMD escaping		ND
CHS	*LYST*	c.5506C > T, p.Arg1836X	Nonsense mutation		NMD		Reduced but detectable
		c.1673dupT, p.Leu558Phefs*22	Frameshift		NMD		Reduced but detectable
		c.5541_5542delAA, p.Arg1848Serfs*3	Frameshift		NMD		Reduced but detectable
		c.3393 + 1G > T	Splicing anomaly				No

*FHL* familial hemophagocytic lymphohistiocytosis, *HPS* Hermansky-Pudlak syndrome, *CGD* chronic granulomatous disease, *WAS* Wiskott-Aldrich syndrome, *XLA* X-linked agammaglobulinemia, *X-SCID* X-linked SCID, *CHS* Chédiak-Higashi syndrome

^a^Aggregated prediction score obtained using Franklin (https://franklin.genoox.com/clinical-db/home)

^b^Nonsense medeated decay (NMD) prediction using Variant Effect Predictor

^c^Protein structure was predicted to be destabilized with a score > 1

### Secondary Effects of IEI Gene Mutations on DBS Protein Profiles

Next, we evaluated the secondary effects of IEI mutations and searched for proteins that could serve as alternative markers for diagnosis/screening of IEIs, particularly those that were unidentifiable by direct evaluation of the causative gene products. For accurate evaluation of DEPs between patients and healthy controls, age-related physiological alterations in protein levels must be taken into account; however, because of their extreme rarity, collection of newborn DBS samples for every IEI is almost impossible. Furthermore, secondary protein profile modification must be considered, since many patient samples were collected after disease onset. Therefore, we searched for possible disease markers by evaluating changes in expression beyond the level of age-dependent variation and secondary modification after disease onset. To this end, we first compared the protein profiles of DBS samples from patients with specific diseases with those from healthy newborns and with those from adults. We then searched for biological pathways that were commonly altered in patient samples relative to both control groups, followed by identification of specific molecules in these pathways that were also in the list of DEPs.

**Fig. 5:**
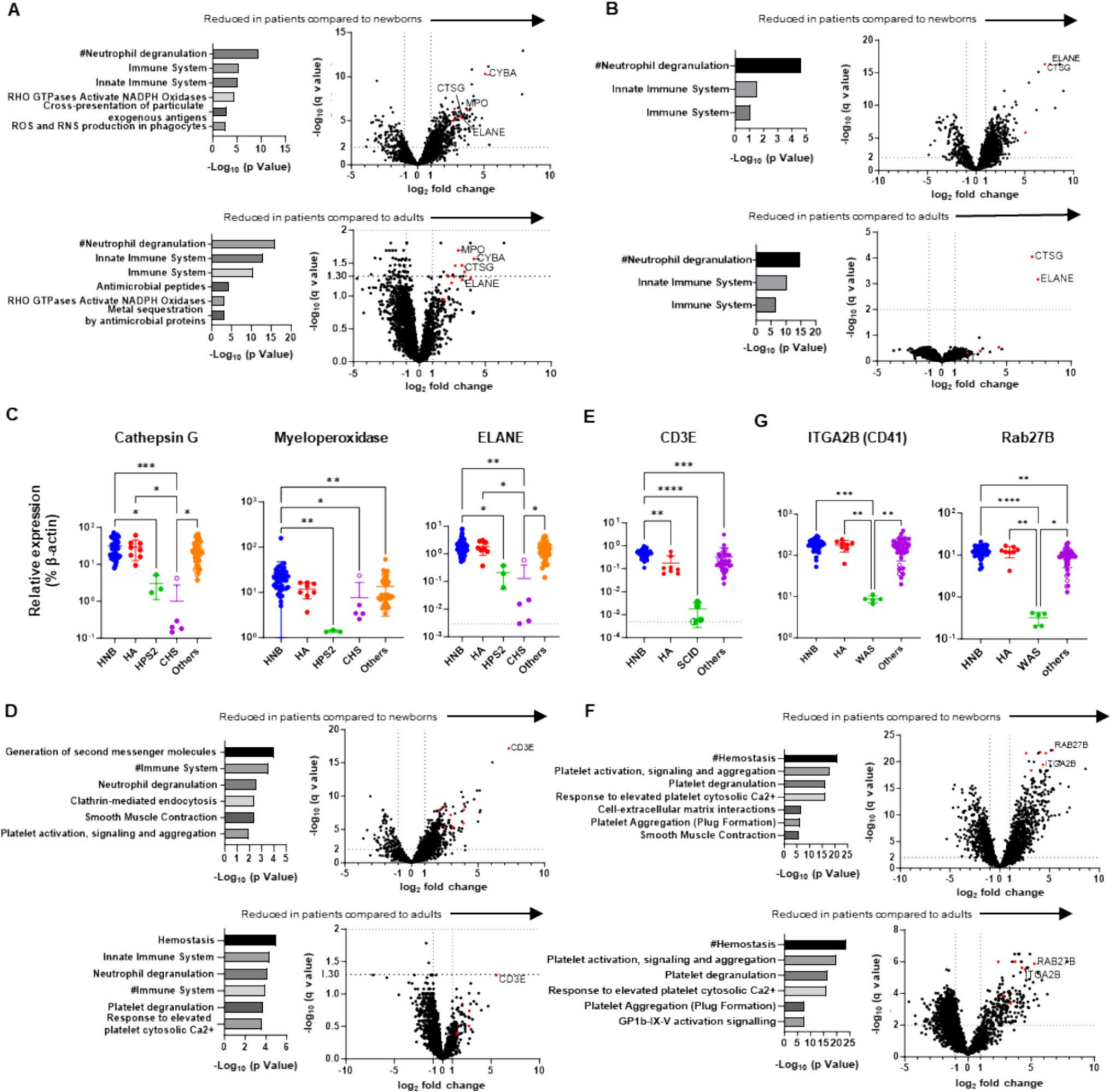
Identification of marker proteins associated with molecular phenotypes of inborn errors of immunity (IEIs). Gene ontology enrichment analysis of differentially expressed proteins (DEPs) (left) and volcano plots showing DEPs (right) in dried blood spot (DBS) samples from patients with **(A)** Hermansky-Pudlak syndrome type 2 (HPS2: *n* = 3), **(B)** Chédiak-Higashi syndrome (CHS: *n* = 5), **(D)** SCID: *n* = 4, and **(F)** Wiskott-Aldrich syndrome (WAS: *n* = 5) relative to samples from healthy newborns (HN) (left) and healthy adults (HA) (right). Levels of **(C)** cathepsin G, myeloperoxidase, and ELANE, **(E)** CD3E, and **(G)** ITGA2B (CD41) and Rab27B in DBS samples from HNB, HA, and patients with IEIs. Statistical differences among groups were analyzed using one-way ANOVA on ranks (the Kruskal–Wallis test), followed by Dunn’s multiple comparisons. Data represent the mean ± SD. *P < 0.05, **P < 0.01, ***P < 0.001, ****P < 0.0001. #, Top Gene Ontology terms commonly altered in patient samples compared to both newborn and adult control groups. Red dots in volcano plots indicate proteins shared in biological pathways commonly altered in patient samples relative to those from HN and HA

The neutrophil degranulation pathway was commonly altered in patients with HPS type 2 relative to healthy newborns and adults, as well as in patients with CHS compared to the two control groups (Fig. 5A and B). Among common DEPs shared in this pathway, levels of p22-phox (*CYBA*), cathepsin G (*CTSG*), and myeloperoxidase (*MPO*), were reduced in samples from patients with HPS type 2, whereas those of cathepsin G and ELANE (*ELANE*) were reduced in patients with CHS relative to controls (Fig. 4F and Fig. 5C). These findings likely reflect the phenotypes of each disease; that is, neutropenia in HPS type 2 and cell granule abnormalities in CHS. By combining ADA and common γ chain deficiencies together as SCID, immune system and neutrophil degranulation pathways were commonly altered in patients with SCID compared to healthy newborns and adults (Fig. 5D). Among common DEPs in these pathways, levels of CD3E were exclusively lower in samples from patients with SCID than in healthy controls (Fig. 5E), likely reflecting the phenotypic alteration in blood cell numbers in this disease (T cell lymphopenia). No protein was found to be common to pathways that were altered in patients with FHL type 2 relative to healthy newborns and adults.

**Fig. 6:**
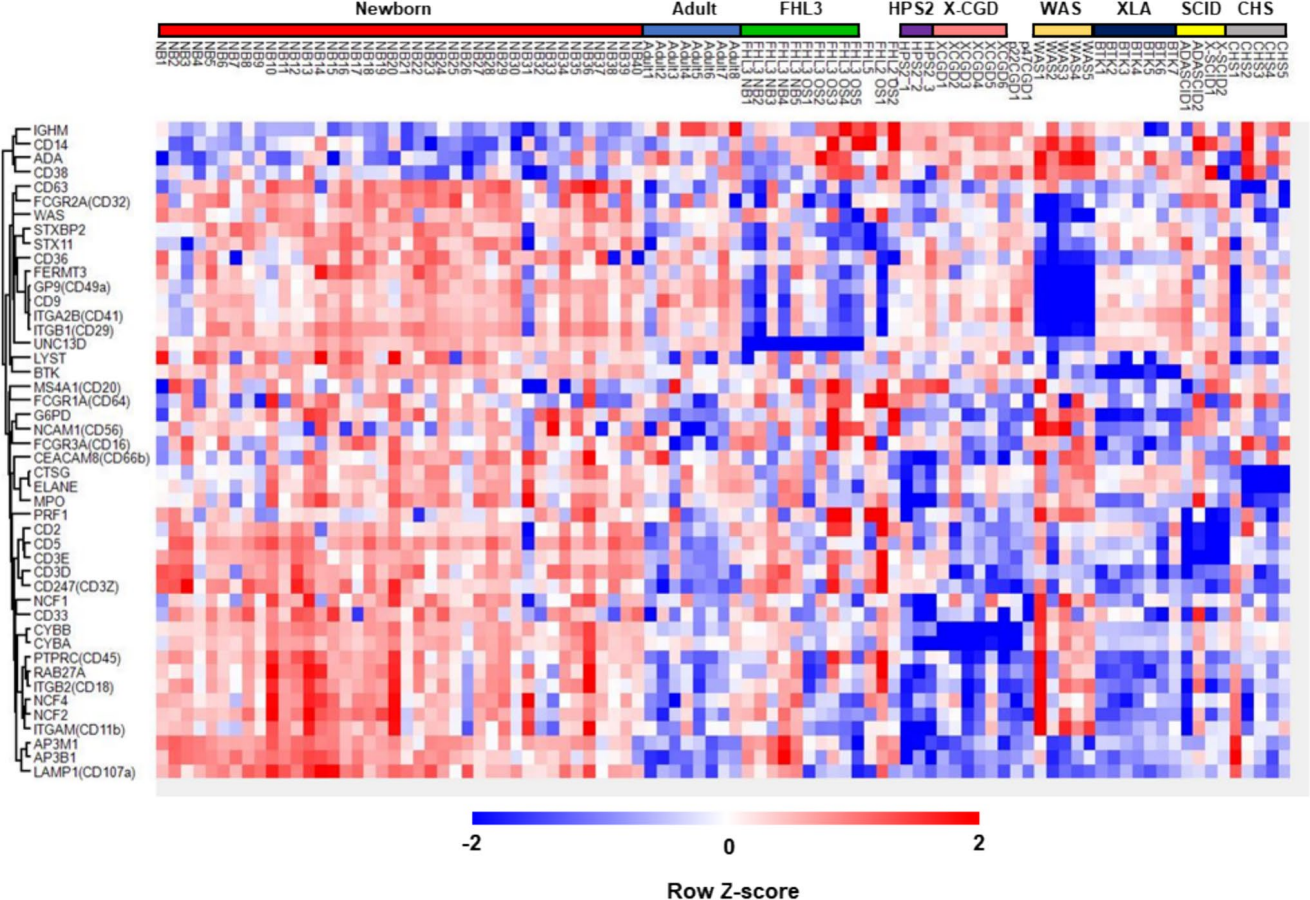
Comparison of proteomics data from patients with inborn errors of immunity with those from healthy newborns and adults. Heatmap showing hierarchical clustering of the levels of 42 proteins that are disease-responsive or associated with molecular phenotypes in dried blood spot samples from healthy newborns, healthy adults, and patients with inborn errors of immunity. Red, relatively higher expression; blue, relatively lower expression

Interestingly, the hemostasis pathway was commonly altered in patients with WAS relative to healthy newborns and adults (Fig. 5G). Among the common DEPs in this pathway, CD41 (*ITGA2B*) and Rab27b (*RAB27B*) were selectively reduced in patients with WAS, likely reflecting thrombocytopenia (Fig. 5H). This explains the reduction of Munc13-4 levels in WAS samples, as Munc13-4 is abundant in platelets (Fig. 5A) [22]. We also found that levels of proteins related to T lymphocytes (CD2, CD3D, CD3E, CD5, CD247), platelets (CD41, CD42b), and neutrophils (CD11b, CD18, CD33, CD66b) were reduced in patients with T cell lymphopenia, thrombocytopenia, and neutropenia, respectively, and could be clustered as distinct groups (Fig. 6), suggesting that assays of these proteins could be used to identify patients with these cell-phenotypical alterations.

## Discussion

Screening for genetic diseases enables early identification of patients who need life-saving treatment before disease onset. The Wilson and Jungner recommendations for population-based screening have guided decisions regarding candidate disease inclusion in NBS programs for the past 50 years; however, the changing landscape of clinical practice and newer technologies, including genome-based methodologies, as well as developing perspectives on the ethical, legal, and social implications of population-based screening practices, necessitate review of these historic criteria [27]. The analysis of multiple genes in newborns raises ethical considerations, and difficulties arise in interpreting variants with yet undetermined significance. The primary purpose of NBS is to convincingly detect significant signs of suspected genetic disease at the pre-symptomatic stage, rather than to conclusively diagnose newborns. Thus, it is reasonable that IEMs have been screened by measuring metabolite levels or activities of metabolic enzymes, as a first-tier test, as abnormal levels of the metabolite or enzymatic activity of interest are directly relevant to IEM pathogenesis; however, suitable markers for other inherited diseases, which can be used to detect functional anomalies at molecular and/or cellular levels before macroscopic disease signs manifest, remain elusive. In this context, we examined the applicability of quantitative protein profiling of DBS, supported by recent progress in DIA-LC–MS/MS technologies, including the development of a simple and reproducible method for pre-treatment of DBS for non-targeted proteome measurements [12].

Using the newly-developed DIA-LC–MS/MS method, we successfully identified and quantified 2912 proteins in all healthy newborn samples tested. Evaluation of these proteins in newborn DBS samples revealed that their profiles were strongly correlated among newborn samples, which clustered distinctly from adult samples (Fig. 1A and B). In agreement with previous reports, newborn DBS contained more fetus-specific proteins (AFP, fetal Hb-related components), as well as proteins related to translational initiation pathways and cell–cell adhesion, and fewer IgA/IgM-related proteins and proteins associated with coagulation and complement pathways (Fig. 2A–D). Of consistently quantified proteins, 1110 were listed in OMIM and 781 were related to diseases with autosomal recessive or X-linked inheritance (Table S2). These included candidate diseases for NBS, such as Menkes disease, Wilson disease, congenital platelet abnormalities, and coagulation factor deficiencies, as well as various IEIs that have potential for screening by detection of reduced levels of disease-responsible proteins.

IEIs are caused by monogenic germline variants that result in loss or gain of function of the encoded proteins, and patients with these conditions present with increased susceptibility to a spectrum of infections, as well as various autoimmune, autoinflammatory, allergic, and/or malignant phenotypes [28]. IEIs are associated with significant morbidity and mortality if left untreated [29]. Patients with severe IEIs require hematopoietic cell transplantation or other curative approaches, with earlier intervention resulting in better outcomes [30–34]. Measuring T cell receptor excision circles (TRECs) and kappa-deleting element recombination circles (KRECs) in DBS samples can identify patients with T and B cell lymphopenias, respectively, and facilitates early treatment intervention and reduction of overall medical costs [27, 35–39]. These results indicate that feasible NBS-based methods to detect other severe IEIs could improve patient outcomes. Furthermore, live vaccines administered in early infancy often cause opportunistic infections in certain groups of infants with IEIs, such as those with SCID and chronic granulomatous disease [40], which could be prevented by NBS.

Our data demonstrate that non-targeted DBS proteomics analysis can detect reduced levels of disease-responsible proteins in samples from patients with FHL type 3, FHL type 5, p91-phox, and p22-phox deficiencies, WAS, and XLA, relative to healthy newborn control samples (Fig. 4A, B, E, F, H, and I). Moreover, the levels of p22-phox and Syntaxin-11 were reduced in parallel with those of p91-phox and Munc18-2, respectively (Fig. 4B–C, E–F), in agreement with previous reports [24, 41]. Munc13-4 protein was specifically decreased in all pre-symptomatic newborn samples from patients with FHL type 3, which had been used for screening for metabolic disorders and stored for months to years, in the same way as detected in post-symptomatic samples (Fig. 4A). These findings demonstrate the robustness of DBS proteomics for NBS of IEIs and also suggest its potential usefulness for diagnosing patients living in regions where other diagnostic modalities are unavailable.

The levels of perforin, LYST, and ADA proteins were not significantly reduced in patients with FHL type 2, CHS, and ADA deficiency, respectively (Fig. 4D, J and L). Many *PRF1* missense mutations result in inappropriate folding or polymerization of perforin molecules, with residual protein expression on western blotting analysis [42]. Two FHL type 2 patients analyzed in this study carried a missense p.Gly220Ser mutation, or an in-frame single amino acid deletion mutation, in one allele. The former missense mutation identified in patient 10 caused a reduction in protein level on western blot analysis with a P1-8 rat anti-perforin antibody raised against amino acid residues 209–380 of mouse perforin [42, 43]. Our proteomics analysis detected six perforin-specific peptides (amino acids 56–65; SGSFPVDTQR, amino acids 88–96; LPLALTNWR, amino acids 288–297; MTASFHQTYR, amino acids 363–371; ALSQYLTDR, amino acids 441–449; LFFGGQELR, and amino acids 465–479; LDFGDVLLATGGPLR), four of which are located outside the amino acid residues identified by the P1-8 antibody. Therefore, although flow cytometry with an antibody that detects polymerized perforin revealed that perforin expression was greatly reduced (Fig. S3), their samples are likely to still contain unpolymerized perforin molecules or residual peptides derived from incompletely degraded perforin protein. Expression of LYST-specific peptides was greatly reduced in the sample from patient 39 (open circle in Fig. 4J), as predicted by in silico analysis. However, nearly normal levels of these peptides were detected in samples from other CHS patients. This is in accordance with a previous report showing that many *LYST* mutations result in residual expression of truncated proteins [44], which cannot be distinguished from full-length LYST on LC–MS/MS, unless a specific peptide near the C-terminus is evaluated. Interestingly, AP3B1 levels varied greatly among patients with HPS type 2, with the highest level in a patient with a splicing site mutation near the 3' end of one allele (Patient #13 in Table 1) and the lowest level in a patient with biallelic nonsense mutations who had experienced an episode of hemophagocytic lymphohistiocytosis (Patient #12 in Table 1) (Fig. 4K), suggesting that HPS type 2 disease severity may correlate with residual AP3B1 expression. Similarly, two ADA deficient patients analyzed in this study carried splice-site and missense mutations, which are more prone to result in higher residual protein levels than nonsense mutations. Furthermore, measurement of ADA by the current method may not be reliable because ADA is predominantly cytosolic and hydrophilic.

As our method was non-targeted, we could also detect disease markers that reflect disease-associated phenotypes. Levels of p22-phox, cathepsin G, and myeloperoxidase were lower in DBS from patients with HPS type 2, and those of cathepsin G and ELANE were lower in patients with CHS, compared with controls (Fig. 5C). These findings reflect the clinical phenotypes of the conditions; neutropenia in patients with HPS type 2 [45], and abnormal azurophil granules in patients with CHS [46]. As p22-phox, ELANE, and cathepsin G are primarily expressed in granulocytes, analyses of these proteins may also be useful in identifying patients with other diseases that are accompanied by neutropenia. CD3E levels were found to be reduced in patients with SCID (Fig. 5E), and those of Rab27B and platelet-related proteins were low in patients with WAS (Fig. 5H), likely reflecting thrombocytopenia and T cell lymphopenia, respectively, in these two IEIs. These results demonstrate that DBS proteomics can detect major changes in the number and/or characteristics of specific cell populations that can currently be analyzed only in specialized laboratories (Fig. 6). Notably, our method could identify patients with T cell lymphopenia and BTK deficiency and may complement TRECs/KRECs analyses.

Another important aspect of the current study is that proteome analysis can provide information more directly relevant to patient conditions than that generated by genetic analysis. As shown in Table 2, the effects of genetic variants on levels of their encoded proteins are often unpredictable using available in silico analysis methods, and interpretation is difficult unless pathogenicity has been reported previously. Non-targeted DBS proteomics can provide in-depth molecular phenotype information and will aid in linking gene structural information with clinical phenotypes.

We acknowledge that this study has several limitations. First, most IEI samples analyzed here were obtained after disease onset, with only six pre-symptomatic neonatal samples included; five from patients with FHL type 3 and one from a patient with CHS. Therefore, further analysis of newborn IEI samples is required to prove the usefulness of the current method for NBS; however, most IEIs analyzed in this study are characterized by significant reductions in expression of disease-related proteins, or in specific cell populations, during the newborn period, which enables the diagnosis of siblings of index patients during their neonatal period before disease onset. In addition, healthy newborns experience physiological leukocytosis, resulting in high expression of leukocyte-related proteins during the neonatal period, and the proteins analyzed in the current study were consistently expressed in all healthy newborn samples. Thus, we do not anticipate that detecting reduced levels of disease marker proteins in newborn samples would be difficult. Second, not all patients display reduced marker protein levels, but protein expression levels often correlate with disease severity [47, 48, 49] and screening for patients with reduced protein levels would be of significant clinical impact. Third, while DIA proteomics analyses using LC–MS/MS can accurately quantify a wide variety of non-target proteins, and adaptation of stable isotope-labeled peptides as internal standards will enable more precise quantification for future practical uses that require more rigorous testing, non-target proteomics is more time consuming than targeted analyses such as monitoring of selected or parallel reactions, and could lead to challenges in providing prompt results for NBS. However rapid advances in MS instrumentation and DIA-LC–MS/MS technology have led to the development of deep non-targeted proteome analysis with short measurement time [50, 51]. Therefore, many patients with IEIs characterized by significant reductions in disease marker proteins could be amenable to screening using non-targeted DIA-LC–MS/MS methods in the near future. Forth, we did not evaluate the unit cost of the test in the current study; the low incidence of severe genetic diseases means that the unit cost of a test is key to its potential for application. The method described in this study can be applied to a wide variety of congenital disorders, including metabolic/neurological and hematological diseases, associated with reduced levels of causative proteins in hydrophobic fractions of peripheral blood cells. Because of the non-targeted approach used, the method may help reveal unknown protein interactions that might advance our understanding of disease pathophysiology. Further, this method can readily be performed in parallel with other screening methods that use the same DBS samples, including TRECs/KRECs analysis and proteome analysis with pre-enrichment of target proteins using specific antibodies. Furthermore, advances in proteomics technology may expand the range of detectable peptides from disease-causing gene products, to allow detection of single amino acid alterations in abundant proteins [52]. Together, such approaches will increase the number of disorders that can be screened simultaneously and reduce the per disease cost.

Every method has advantages and disadvantages, and no single test can screen for every genetic disease with high accuracy and efficiency. Screening of autosomal recessive disorders by gene sequencing is hampered due to difficulty in determining the cis/trans state of identified mutations. In addition, the results of whole exome/genome-wide sequencing cannot be linked to clinical phenotypes in the absence of accumulated in-depth genotype–phenotype data. Proteomics-based testing and TRECs/KRECs analysis can be performed together with whole exome/genome-wide sequencing using the same DBS sample, providing phenotypic information to support the diagnosis. Although additional improvements are needed for population-based applications, methodological refinements and innovations will enable the use of this approach to screen newborns for various diseases and strengthen the application of genome-wide sequencing-based screening in the future [27, 53].

## Conclusions

Our findings demonstrate that levels of multiple proteins associated with genetic disorders in DBS samples can be semi-quantified, with no need for pre-enrichment using specific antibodies. Proteomic analysis of DBS samples can serve as a “single-platform-to-assess-many-diseases” NBS approach for IEIs and various other genetic disorders, enabling the identification of patients before disease onset, allowing earlier intervention in the absence of severe complications, and improving patient prognosis.

## Supplementary Information

Below is the link to the electronic supplementary material.

Supplementary file1 (DOCX 416 KB)

Supplementary file2 (XLSX 3212 KB)

Supplementary file3 (XLSX 78 KB)

## Data Availability

Table S1 and S2 are also available at 10.5281/zenodo.13890745. All figures were generated using the data in Table S1.
