# Supplementary material for: A Non-targeted Proteomics Newborn Screening Platform for Inborn Errors of Immunity
Source: J Clin Immunol. 2024 Oct 25;45(1):33. doi: 10.1007/s10875-024-01821-7 (PMC11511704; doi:10.1007/s10875-024-01821-7)
Supplement: Supplementary file 1 — Supplementary file1 (DOCX 416 KB) [file 10875_2024_1821_MOESM1_ESM.docx]

**Supplemental Figures**

**A Non-targeted Proteomics Newborn Screening Platform for Inborn Errors of Immunity**

Hirofumi Shibata, Daisuke Nakajima, Ryo Konno, Atsushi Hijikata, Motoko Higashiguchi,

Hiroshi Nihira, Saeko Shimodera, Takayuki Miyamoto, Masahiko Nishitani-Isa, Eitaro Hiejima,

Kazushi Izawa, Junko Takita, Toshio Heike, Ken Okamura, Hidenori Ohnishi, Masataka Ishimura,

Satoshi Okada, Motoi Yamashita, Tomohiro Morio, Hirokazu Kanegane, Kohsuke Imai,

Yasuko Nakamura, Shigeaki Nonoyama, Toru Uchiyama, Masafumi Onodera, Ryuta Nishikomori,

Osamu Ohara, Yusuke Kawashima, and Takahiro Yasumi

**
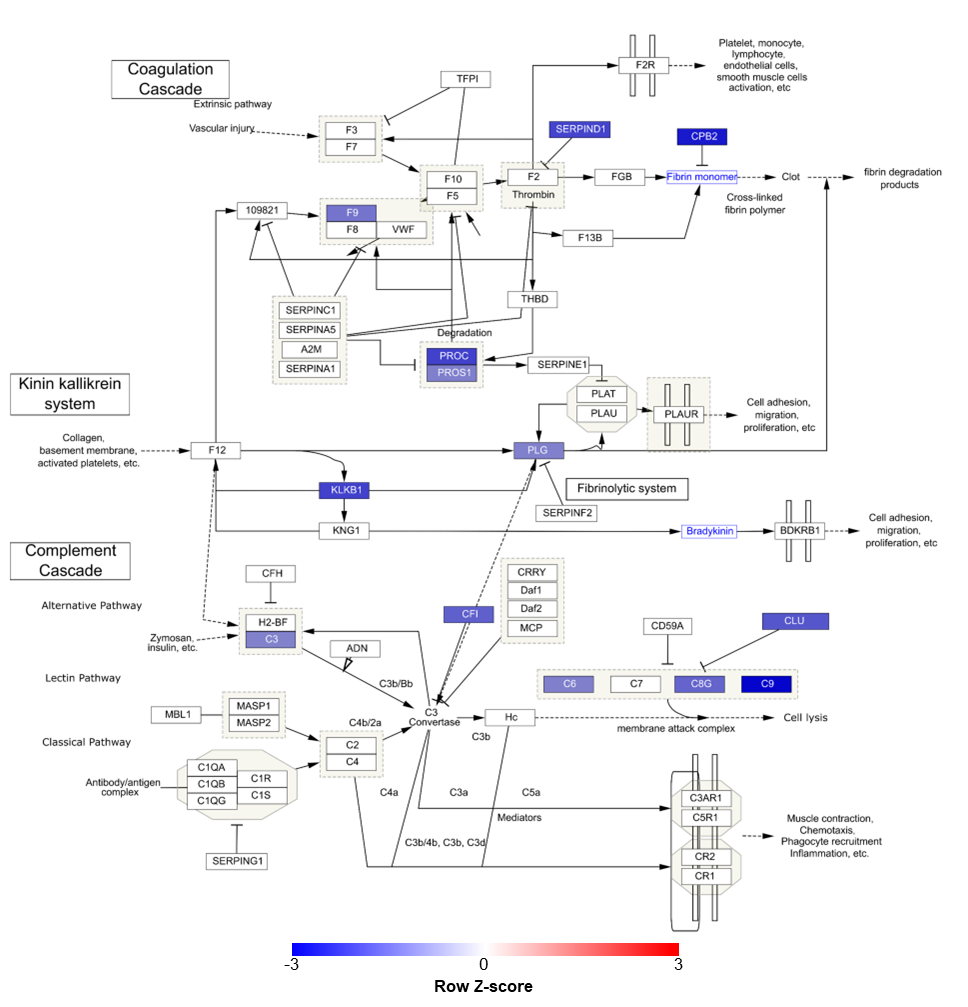
**

**Fig. S1. Wikipathway map of the Complement and coagulation cascades.**

Pathways were retrieved and visualized from WikiPathways using Cytoscape, highlighting downregulated proteins (P < 0.05) in healthy newborns relative to adults. Blue node colors denote differentially expressed proteins and fold changes in their expression. Blue characters indicate metabolite molecules.

**
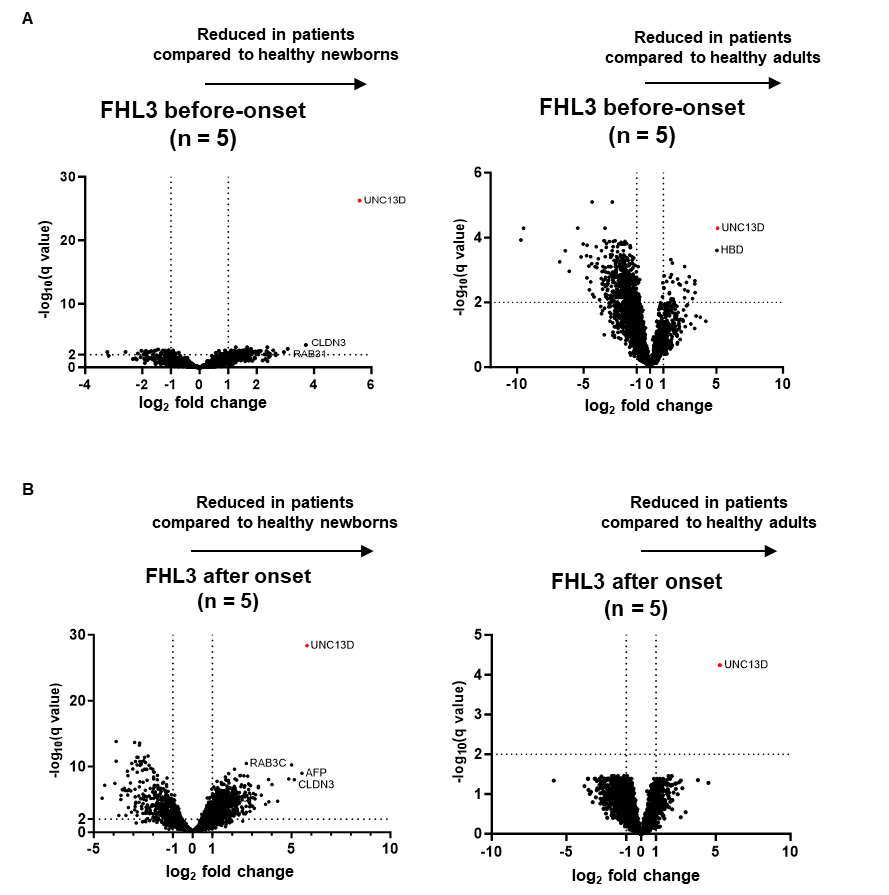
**

**Fig. S2. Munc13-4 is conspicuously reduced in dried blood spot (DBS) samples from patients with familial hemophagocytic lymphohistiocytosis type 3 (FHL3).**

Volcano plots showing the differentially expressed proteins in DBS samples from patients with FHL3 that were obtained **(A)** before and **(B)** after disease onset compared to samples from healthy newborns (left) and from healthy adults (right).

**
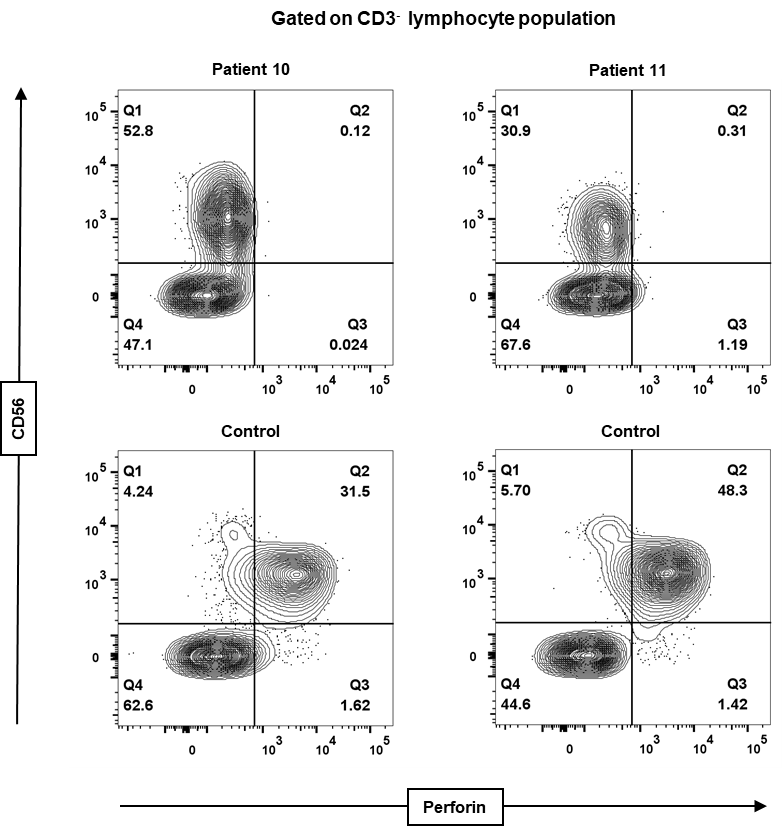
**

**Fig. S3. Flow cytometric analysis of perforin expression in natural killer cells from patients with familial hemophagocytic lymphohistiocytosis type 2.**

Fluorescence-activated cytometric analysis of perforin expression in CD3^–^ peripheral lymphocytes obtained from patients 10 and 11 relative to healthy controls. Peripheral blood mononuclear cells were stained with fluorescein isothiocyanate (FITC)-conjugated anti-CD3 (SK7, eBioscience), Brilliant Violet (BV) 510-conjugated anti-CD16 (3G8, BioLegend), and allophycocyanin (APC)-conjugated anti-CD56 (N901, Beckman Coulter) monoclonal antibodies and then incubated for 20 min. Next, the cells were fixed and permeabilized with Cytofix/Cytoperm (BD Biosciences) and then stained with a PE-conjugated anti-perforin antibody (δG9, eBioscience) or an isotype control antibody.
